# Supplementary material for: Screening for Preeclampsia and Fetal Growth Restriction in the First Trimester in Women without Chronic Hypertension
Source: J Clin Med. 2023 Aug 27;12(17):5582. doi: 10.3390/jcm12175582 (PMC10488103; doi:10.3390/jcm12175582)
Supplement: Supplementary file 1 [file jcm-12-05582-s001.zip › jcm-2527667-supplementary.pdf]

Table S1. Correlations between first trimester biophysical and biochemical markers and selected perinatal parameters among groups at high risk for PE or FGR or those who developed PE or FGR.

Group	PE diagnosis n=19		without PE n=855		FGR or SGA diagnosis n= 51		without FGR or SGA n=823	
Feature	Birth week	Birth weight	Birth week	Birth weight	Birth week	Birth weight	Birth week	Birth weight
	R, p	R, p	R, p	R, p	R, p	R, p	R, p	R, p
MoM UtPI	0.37, 0.13	0.17, 0.49	**0.08, 0.03**	-0.03, 0.4	-0.06, 0.69	-0.17, 0.24	**0.09, 0.009**	-0.01, 0.87
MoM PAPP-A	-0.25, 0.3	0.14, 0.55	0.06, 0.08	**0.15, <0.001**	0.21, 0.13	0.22, 0.13	0.03, 0.4	**0.13, <0.001**
MoM PLGF	-0.04, 0.88	-0.02, 0.94	0.02, 0.47	**0.1, 0.004**	0.19, 0.19	0.18, 0.21	0.01, 0.79	0.07, 0.051
MoM MAP	-0.41, 0.09	-0.29, 0.22	0.04, 0.32	-0.03, 0.47	0.09, 0.54	0.08, 0.58	0.01, 0.69	-0.05, 0.15
Group	High risk for PE n= 35		Low risk for PE n= 839		High risk for FGR n=74		Low risk for FGR n=800	
Feature	Birth week	Birth weight	Birth week	Birth weight	Birth week	Birth weight	Birth week	Birth weight
	R, p	R, p	R, p	R, p	R, p	R, p	R, p	R, p
MoM UtPI	0.01, 0.96	0.11, 0.54	0.07, 0.051	-0.03, 0.35	0.13, 0.3	0.09, 0.47	**0.08, 0.01**	-0.01, 0.77
MoM PAPP-A	0.22, 0.21	0.28, 0.11	0.05, 0.16	**0.14, <0.001**	0.18, 0.1	**0.27, 0.02**	0.04, 0.31	**0.12, <0.001**
MoM PLGF	0.1, 0.55	0.07, 0.68	0.03, 0.39	**0.09, 0.01**	-0.04, 0.7	0.04, 0.73	0.02, 0.61	0.06, 0.08
MoM MAP	0.11, 0.52	- 0.04, 0.83	0.02, 0.57	-0.03, 0.45	-0.07, 0.57	-0.03, 0.78	0.03, 0.39	-0.03, 0.46

Group	High risk for PE or/and FGR n=81		Low risk for PE and FGR n=793	
Feature	Birth week	Birth weight	Birth week	Birth weight
	R, p	R, p	R, p	R, p
MoM UtPI	0.11, 0.33	0.08, 0.49	**0.08, 0.02**	-0.01, 0.71
MoM PAPP-A	0.2, 0.08	**0.3, 0.006**	0.04, 0.31	**0.11, 0.001**
MoM PLGF	-0.01, 0.9	0.09, 0.43	0.02, 0.59	0.06, 0.09
MoM MAP	0.001, 0.99	0.06, 0.62	0.03, 0.43	-0.03, 0.4

Note: PE: preeclampsia; FGR: fetal growth restriction; MoM: multiple of the median; UtPI: uterine artery pulsatility index; PAPP-A: Pregnancy Associated Plasma Protein-A; PLGF: placental growth factor;
